# The neural basis of face pareidolia with human intracerebral recordings

**DOI:** 10.1162/imag_a_00518

**Published:** 2025-03-31

**Authors:** Begüm Cerrahoğlu, Corentin Jacques, Diane Rekow, Jacques Jonas, Sophie Colnat-Coulbois, Stephanie Caharel, Arnaud Leleu, Bruno Rossion

**Affiliations:** Université de Lorraine, InterPsy, Nancy, France; Université de Lorraine, CNRS, IMoPA, Nancy, France; Biological Psychology and Neuropsychology, University of Hamburg, Hamburg, Germany; Université de Lorraine, CHRU-Nancy, Service de Neurologie, Nancy, France; Université de Lorraine, CHRU-Nancy, Service de Neurochirurgie, Nancy, France; Development of Olfactory Communication and Cognition Lab, Centre des Sciences du Goût et de l’Alimentation, Université Bourgogne Europe, CNRS, INRAe, Institut Agro, Dijon, France

**Keywords:** face pareidolia, intracerebral recordings, frequency tagging, ventral occipito-temporal cortex, facelike objects

## Abstract

The perception of a meaningful facial pattern on a nebulous stimulus—face pareidolia—is a typical human experience. Neuroimaging and electrophysiological studies have generally shown similarities in the spatio-temporal responses to typical faces and objects eliciting face pareidolia, that is, facelike objects. However, the extent to which facelike objects engage the same neural basis as human faces remains unclear. To address this issue, we used direct measures of brain activity from intracerebral electrodes implanted in the ventral occipito-temporal cortex (VOTC) of a large group of patients (n = 44). Face selectivity was determined by contrasting a large set of naturalistic face or facelike object images with non-face object categories. High signal-to-noise ratio face-selective and facelike object-selective responses were objectively identified and quantified with frequency tagging and compared in space and time throughout the VOTC. Selective activity to facelike objects was found in all key regions of the human cortical face network, extending to the previously unexplored anterior temporal lobe (ATL). Although category-selective activity was markedly reduced for facelike objects compared with human faces, consistent with previous findings, 89% of facelike object-selective contacts spatially overlapped with human face-selective contacts, while the remaining spatially scattered contacts recorded negligible responses. Furthermore, the amplitude of the two face-selective neural signals showed high correlations across regions, recording contacts and time courses as well as concurrent early onset, challenging the view that facelike objects are interpreted as faces through feedback from higher order brain regions. Together, our findings demonstrate that the pareidolic perception of face in facelike objects engages the same ventro-temporal neural circuitry, with the same temporal dynamics, as human faces.

## 1 Introduction

Pareidolia is defined as the perception of a meaningful pattern in nebulous stimuli, often visual in nature. It is a highly common experience of the human perceptual system, in particular for faces (i.e., “face pareidolia”;Zhou & Meng, 2021). In specific cases of face pareidolia, highly recognizable stimuli, such as everyday living and non-living objects, induce the perception of a face despite the absence of biological facial features (e.g., the face on the moon, Jesus on toast, Arcimboldo’s paintings;Everitt, 2013;Healy, 2009;Hulten, 1987). Objects that trigger this phenomenon are typically referred to as*facelike objects*.

Face pareidolia begins at an early age, being present already in 3–5-year olds (Beran et al., 2017) or even earlier (seeRekow et al., 2021), and increases in occurrence over time with age until late adulthood (Flessert et al., 2023;Rahman & van Boxtel, 2022;Wardle et al., 2023). There is mixed evidence regarding whether other animal species, including non-human primates, experience face pareidolia, with more recent evidence suggesting that face pareidolia, as defined here, is human specific (Flessert et al., 2023;Koyano et al., 2025;Sharma et al., 2023;Tomonaga & Kawakami, 2022).

A specific interest of pareidolic face stimuli lies in their visual diversity, allowing to study human face perception with stimuli that diverge from typical biological facial features (skin color, round shape, etc.). Moreover, despite their physical variability, facelike features and configurations can be swiftly perceived from everyday objects, suggesting that (face) perception relies on broadly tuned memory representations that are sensitive to diverse (facelike) features and their spatial organization. Another interesting feature of facelike objects that give rise to face pareidolia is that these stimuli challenge conventional category membership since the perception of a face does not negate the perception of the same stimuli as objects (Stuart et al., 2023). Overall, the phenomenon of face pareidolia, in particular for facelike objects, provides a valuable tool for experimental exploration into human face perception and its neural basis.

However, the extent to which face pareidolia engages the same processes and neural circuits as human faces remains unclear. On the one hand, face pareidolia shares many characteristics with natural face perception, whether it arises from meaningless stimuli or objects. For instance, face pareidolia typically arises rapidly (Ariga & Arihara, 2017;Keys et al., 2021;Stuart et al., 2023) and spontaneously, that is, without any conscious effort (Rekow et al., 2022a;Saurels et al., 2024). Also, similarly to human faces (Golan et al., 2014;Hershler & Hochstein, 2005,^2006^,^2010^;Langton et al., 2008;Mayer et al., 2015;Purcell & Stewart, 1988;VanRullen, 2006), pareidolic faces appear to be attentionally privileged over objects without faces, leading to a visual search advantage (Caruana & Seymour, 2021;Jakobsen et al., 2023;Keys et al., 2021;Zhou & Meng, 2021), which persists over the periphery (Takahashi & Watanabe, 2015, but seeSaurels et al., 2024). Additionally, pareidolic faces present other behavioral effects often associated with human faces, such as gaze cueing (Palmer & Clifford, 2020;Ristic & Kingstone, 2005;Takahashi & Watanabe, 2013), improved individuation abilities (Vuong et al., 2017), emotion detection (Lipp & Taubert, 2024;Wardle et al., 2022), and serial dependence of perceived expression (Alais et al., 2021), though these effects are less pronounced than with human faces.

In line with these behavioral observations, studies using electro- or magneto-encephalography (EEG/MEG) have reported an early neural response (N170/M170) to facelike objects similar in timing and spatial location to human faces, whereas non-face objects elicit lower N170/M170 peak amplitudes (Caharel et al., 2013;Churches et al., 2009;Proverbio & Galli, 2016; seeRossion, 2014for review; see alsoRekow et al., 2022a,2022bfor EEG face-selective responses measured with frequency tagging). MEG and fMRI studies have also suggested sources in face-selective regions of the ventral occipito-temporal cortex (VOTC) for these early neural responses to pareidolic faces (Akdeniz et al., 2018;Boccia et al., 2015;Decramer et al., 2021;Hadjikhani et al., 2009;Romagnano et al., 2024;Wardle et al., 2017,^2020^). Neural activation for facelike objects is generally lower than for biological faces but higher than for non-face objects (Decramer et al., 2021;Wardle et al., 2017,^2020^). Such diminished responsiveness could have several non-mutually exclusive causes. On the one hand, the responses to facelike objects and human faces might be generated by the same face categorization network in the VOTC, but with weaker or less consistent activation for facelike objects compared with human faces due to visual difference between the two categories (e.g., missing diagnostic human facial cues; higher visual similarity between facelike and non-face objects than between facelike objects and human faces). Alternatively, only a subset of face-selective regions may generate the facelike categorization response (possibly predominantly within the right hemisphere;Caharel et al., 2013;Proverbio & Galli, 2016;Rekow et al., 2022a,2022b).

While fMRI offers significant advantages such as non-invasiveness and high spatial resolution, it is also limited in its investigation of the neural basis of face pareidolia. One challenge lies in its indirect measurement of neural activity, leading to significant fluctuations in signal-to-noise ratio (SNR) across brain regions. In particular, exploring regions anterior to the middle fusiform gyrus, in the ventral temporal lobe, is limited by significant magnetic susceptibility artifacts from the ear canals (Wandell, 2011;Rossion et al., 2024). Consequently, even with corrected-distortion fMRI sequences (e.g.,Embleton et al., 2010;Hoffman & Lambon Ralph, 2018), these neuroimaging studies cannot fully identify and compare face-selective neural activity across the VOTC (Collins et al., 2016;Rice et al., 2018; see discussion inRossion et al., 2018,^2024^). Additionally, fMRI studies in this area of research focus on specific regions of interest or local response patterns, hindering the ability to assess the extent to which facelike object processing engages the whole cortical face network, especially anterior VOTC regions. In one illustrative example, a searchlight decoding analysis utilizing fMRI data to assess decoding accuracy in discriminating faces from non-face objects as well as facelike objects from non-face objects suggests the absence of distinguishable responses in the VOTC beyond the “fusiform face area” and the “occipital face area” (FFA and OFA, respectively), with some intermediary regions bridging these areas (seefigure 3inWardle et al., 2020).

Here we provide an original contribution to understanding the neural basis of face pareidolia in the human brain, exploring the extent to which it involves the ventral human face categorization network with direct recordings of neural activity. To this end, we measured intracerebral electrophysiological neural signals from recording sites in the VOTC of a large number of epileptic patients (n = 44; 2,695 recording contacts in the VOTC gray matter). We employed a frequency-tagging visual stimulation paradigm, optimized for assessing categorical selectivity for faces and facelike objects, as previously validated in EEG, and which enables the objective definition and quantification of differential responses to human faces and facelike objects compared with non-face objects in the frequency domain with a high SNR (Rekow et al., 2022a,2022b).

We hypothesize that the selective responses to human face and facelike objects are generated from the same face-selective regions distributed along the VOTC, but with a weaker overall neural activity for facelike objects, in line with findings from other approaches (Churches et al., 2009;Decramer et al., 2021;Rekow et al., 2022a,2022b;Wardle et al., 2017,^2020^). Alternatively, it could also be that only a subset of face-selective regions is responsible for the facelike categorization response, potentially predominantly within the right hemisphere (Rekow et al., 2022a). This would suggest that the reduced responsiveness measured at a distance (on the scalp) is due to fewer or different neural sources being dedicated to processing facelike objects, rather than the same network processing them less efficiently. Of particular interest in the present study is the (ventral) anterior temporal lobe (vATL), which plays a key role in semantic memory (Lambon Ralph & Patterson, 2008;Rice et al., 2015), in particular for person-related semantics (e.g.,Blank et al., 2014;Rice et al., 2018;Collins et al., 2016). One possibility is that face-selective activity extending to regions of the vATL down to the temporal pole (Jonas et al., 2016;Jacques et al., 2022) is limited to real human faces, which, unlike facelike objects, are more strongly associated with a rich network of semantic information. Given the wide sampling of neural activity in the ATL afforded by intracerebral recordings, the present study is particularly well suited to test this hypothesis.

## 2 Materials and Methods

### 2.1 Participants

**Fig. 1.**
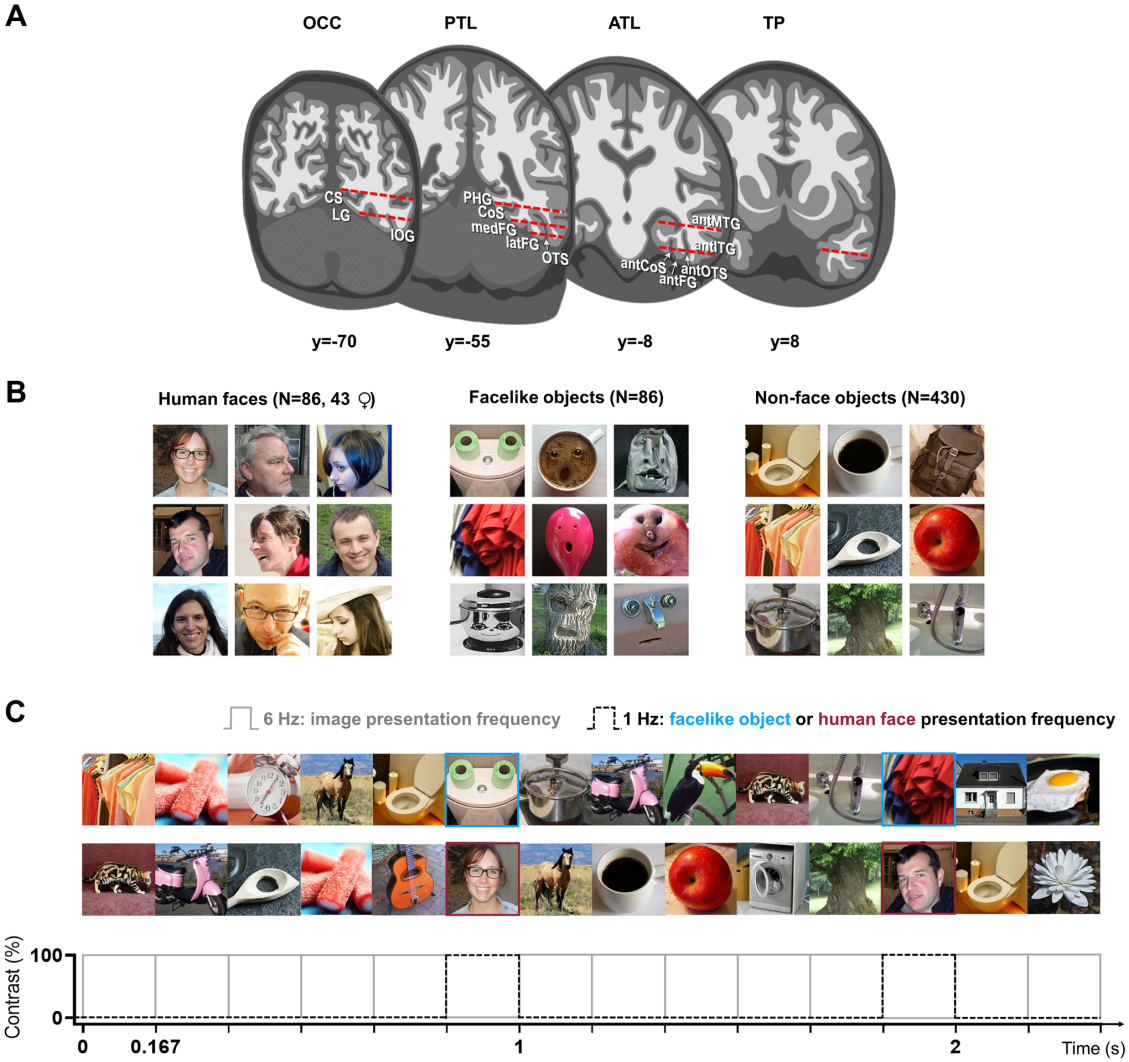
SEEG methods and fast periodic visual stimulation (FPVS) paradigm. (A) Schematic coronal representation of the typical trajectories of depth electrodes implanted in the VOTC across patients (adapted fromJonas et al., 2016). Each electrode contains 8–15 contiguous recording contacts (red dashes) spread along the electrode length on the medio-lateral axis. OCC: occipital lobe; CS: calcarine sulcus; LG: lingual gyrus; IOG: inferior occipital gyrus; PTL: posterior temporal lobe; PHG: parahippocampal gyrus; COS: collateral sulcus; medFG: medial fusiform gyrus; latFG: lateral fusiform gyrus; OTS: occipito-temporal sulcus; ATL: anterior temporal lobe; antMTG: anterior middle temporal gyrus; antITG: anterior inferior temporal gyrus; antCOS: anterior collateral sulcus; antOTS: anterior occipito-temporal sulcus; antFG: anterior fusiform gyrus. (B) Examples of variable natural images of human faces, facelike objects, and non-face objects used as stimuli (adapted fromRekow et al., 2022a). (C) Example of ≈2.3 s of visual stimulation at 6 Hz (i.e., 6 images per second, ≈167 ms per image cycle) without an inter-stimulus interval (adapted fromRekow et al., 2022a). In separate sequences, facelike objects or human faces are periodically inserted at 1 Hz (every 6th stimulus).

The study included 44 participants (20 females, mean age = 34.6 ± 9.4 years; 42 right handed) undergoing clinical intracerebral evaluation with depth electrodes (stereotactic electroencephalography or SEEG,Fig. 1A) for refractory partial epilepsy. The implantation of intracerebral electrodes was performed solely for clinical purposes, as part of presurgical evaluation to identify seizure foci. Participation in the study was entirely voluntary and independent of their clinical care. All participants provided written consent to take part in the study, which was conducted according to the principles expressed in the declaration of Helsinki and approved by a national ethics committee certified by the French Ministry of Health (Institutional Review Board: IORG0009855). Participants were included in the study if they had at least one intracerebral electrode implanted in the VOTC. While patients with refractory partial temporal epilepsy can be impaired and/or slowed down at explicit tasks requiring to recognize familiar faces or encode new faces in memory (Milner, 1968;Seidenberg et al., 2002;Drane et al., 2008,^2013^), their ability to process pictures of unfamiliar faces and to recognize even degraded visual stimuli (e.g., Mooney faces) as faces appears to be largely unimpaired (Volfart et al., 2020).

### 2.2 Intracerebral electrode implantation and SEEG recording

Intracerebral electrodes (Dixi Medical, Besançon, France) were stereotactically implanted within the participants’ brains for clinical purposes, that is, to delineate their seizure onset zones (Talairach & Bancaud, 1973) and to functionally map the surrounding cortex for potential epilepsy surgery (Bédos Ulvin et al., 2017). Each intracerebral electrode, with a diameter of 0.8 mm, contains 5 to 15 independent recording contacts, each 2 mm in length, spaced 1.5 mm apart from edge to edge (seeSalado et al., 2017, for details about the electrode implantation procedure). Given the small diameter of the electrodes (0.8 mm), the damage to brain tissue from implantation is minimal and does not cause large-scale damage to healthy tissue (seeCampbell & Wu, 2018for a review). The exact anatomical location of each recording contact was determined by co-registration of post-operative non-stereotactic CT-scan with a pre-operative T1-weighted MRI.

A total of 602 electrode arrays were implanted in the VOTC of the 44 participants. These electrodes contained 2,695 individual recording contacts in the VOTC (i.e., in the gray matter or medial temporal lobe-MTL; 1,626 contacts in the left hemisphere, 1,069 in the right hemisphere). SEEG was sampled at either 500 or 512 Hz and referenced to either a midline prefrontal scalp electrode (FPz, 36 participants) or an intracerebral contact in the white matter (in 8 participants), depending on the clinical availability of a scalp electrode. The SEEG signal at each recording contact was re-referenced offline to a bipolar reference in order to mitigate dependencies between neighboring contacts (Li et al., 2018). Specifically, the signal at each recording contact was calculated by subtracting the signal measured at that contact (i.e., using the original recording reference) from the signal measured at the immediately adjacent contact positioned more medially on the same SEEG electrode array. As SEEG field potentials are derived from pairs of adjacent contacts, each electrode array provides one fewer contact for analysis compared with the initial set of recording contacts. All subsequent analyses were conducted on the bipolar-referenced signal within the set of bipolar contacts elicited by the method described above.

### 2.3 Fast periodic visual stimulation paradigm

Categorical selectivity for both human faces and facelike objects was measured using a frequency-tagging visual stimulation paradigm which has been previously validated using scalp EEG (Rekow et al., 2022a,2022b).

#### 2.3.1 Stimuli

The stimuli consisted of natural color images, including 86 human faces (43 females), 86 facelike objects, and 430 non-face objects, all cropped to a square format and resized to 300 × 300 pixels. Each item depicted retained its original background and was intentionally varied in size, viewpoint, lighting, contrast, and background to ensure a broad range of physical characteristics (examples shown inFig. 1B). The non-face objects included various biological and manufactured items, each with multiple exemplars (ranging from 3 to 20) per category. Additionally, human faces exhibited substantial diversity in age, sex, race, and expression. As a result of this variability in low-level visual cues associated with both faces and objects, the influence of these cues on the recorded selective neural responses is limited (Gao et al., 2018;Rossion et al., 2015).

The facelike stimuli used here were selected byRekow et al. (2022a)from a set of 224 facelike object images obtained from the internet, based on their facelikeness as assessed in a pretest. Importantly, facelike object images depicted a range of object categories (with 1 to 5 exemplars in each category), several of which matched the non-face object categories such that facelike objects differed from non-face objects solely in their overall appearance resembling faces (seeRekow et al., 2022afor a detailed description of the paradigm and the stimuli set). Stimuli were presented at the center of a 24-inch LED screen (1,920 × 1,080 resolution, 120 Hz refresh rate) on a mid-gray background (128/255 grayscale) and at 80 cm distance subtended approximately 7.9° of visual angle.

#### 2.3.2 Procedure

Participants viewed continuous sequences of images presented at a fast rate of 6 Hz (i.e., 6 images per second, ≈167 ms per image cycle) without inter-stimulus interval (forward masked and backward masked) using a square wave contrast modulation (Fig. 1C). This relatively fast rate allows only one fixation per stimulus, with a stimulus duration that is largely sufficient to elicit maximal face-selective activity (Retter & Rossion, 2016).

In separate sequences, depending on the condition, images of human faces or facelike objects appeared periodically every 6th stimulus (i.e., at 6/6 = 1 Hz), while the remaining base images consisted of the same set of non-face objects. This frequency tagging approach ensures that neural activity common to both human faces or facelike objects and non-face stimuli—elicited at every image cycle—is expressed at 6 Hz and its harmonics. In contrast, if the oddball stimuli generate a differential (i.e., selective) response to the periodic oddball category (human faces or facelike objects) compared with successive non-face objects, this response is expressed at the exact oddball frequency of 1 Hz (i.e., 6 Hz/6) and its harmonics (Fig. 1B). All images were randomly selected from their respective categories. Repetition began after all unique images had been shown, and no image could be immediately repeated. A stimulation sequence lasted 70 s: 66 s of stimulation at full contrast flanked by 2 s of fade-in and fade-out, where contrast gradually increased or decreased (between 0% and 100%), respectively.

During a sequence, participants were instructed to fixate a small black cross which was presented continuously at the center of the stimuli, and to detect brief (500 ms) color changes (black to red) of this fixation cross. Each participant viewed two stimulation sequences each for the human face and facelike object oddball conditions (i.e., 4 x 70 s overall).

### 2.4 SEEG signal processing and analyses

SEEG signal processing in the frequency domain followed a pipeline established by previous studies using this approach (Hagen et al., 2020;Jacques et al., 2020,^2022^;Jonas et al., 2016;Lochy et al., 2018). Segments of SEEG corresponding to the stimulation sequences were extracted (74-s segments, -2 s to +72 s). The 74-s data segments were then cropped to contain an integer number of 1 Hz cycles, starting 2 s after the onset of the sequence (immediately after the fade-in period) and continuing until approximately 68 s, just before stimulus fade-out (66 cycles of human face or facelike object stimuli ≈65.8 s). Segments were averaged in the time domain, and a fast Fourier transform (FFT) was then applied to these averaged segments, and amplitude spectra were extracted for all recording contacts. The frequency-tagging approach used here allows objectively identifying and separating two distinct types of responses (Jonas et al., 2016): (1) a general visual response occurring at the base stimulation frequency (6 Hz) and its harmonics and (2) a human face- or facelike object-selective activity at 1 Hz and its harmonics. Importantly, since (S)EEG noise is broadband and these responses (i.e., the signal) concentrate in small frequency bins defined by the high spectral resolution (i.e., 1/65.6 s = 0.0152 Hz), signal-to-noise ratio is very high (Regan, 1989).

Human face- and facelike object-selective activities significantly above noise level at their predefined stimulation frequency (1 Hz and harmonics) were determined as follows (e.g.,Lochy et al., 2018;Jacques et al., 2020,^2022^): (1) the FFT spectrum was cut into five segments centered at the human face/facelike object presentation frequency and harmonics (1, 2, 3, 4, 5 Hz) and surrounded by 25 neighboring bins on each side; (2) the amplitude values in these five segments of FFT spectra were summed; (3) the summed FFT spectrum was transformed into a z-score. Z-scores were computed as the difference between the amplitude at the human face or facelike object frequency bin and the mean amplitude of 48 surrounding bins (25 bins on each side, excluding the 2 bins directly adjacent to the bin of interest) divided by the standard deviation of amplitudes in the corresponding 48 surrounding bins. A contact was considered as showing a human face- and/or facelike object-selective response if the*z*-score at the frequency bin of their stimulation exceeded 3.1 (i.e.,*p*< .001, one-tailed: signal > noise).

Based on the discrimination response patterns observed across the two conditions, we categorized significant contacts as follows: (1) Contacts displaying a significant selective response to human faces but not to facelike objects were labeled as “human face only” (human face+ only); (2) contacts displaying a significant selective response to facelike objects but not to human faces were labeled as “facelike object only” (facelike object+ only); (3) contacts displaying a significant selective response to both human faces and facelike objects were labeled as “overlap” (human face+ facelike object+).

### 2.5 Quantification and analysis of response amplitudes

Baseline-subtracted amplitudes were computed in the frequency domain as the difference between the amplitude at each frequency bin and the average of 48 corresponding surrounding bins (up to 25 bins on each side, i.e., 50 bins, excluding the 2 bins directly adjacent to the bin of interest, i.e., 48 bins). For each contact, human face-selective and facelike object-selective amplitudes were quantified as the sum of the baseline-subtracted amplitudes at the oddball frequency from the 1st until the 15th harmonic (1 until 17 Hz), excluding the 6th and 12th harmonics (6 and 12 Hz) that coincided with the base frequency. This summation range of harmonics was constrained by the highest significant harmonic identified across participants (*z*-score > 3.1;*p*< .001). Base response amplitudes were quantified separately for the human face and facelike object sequences as the sum of the baseline-subtracted amplitudes at the base frequency from the 1st until the 4th harmonic (6 until 24 Hz), with no significant base frequency responses found above the 4th harmonic across participants. Prior to performing statistical comparisons of mean amplitudes across anatomical regions and hemispheres, amplitudes from each anatomical region and/or hemisphere were winsorized separately (i.e., normalized by clipping the largest and smallest 10% of amplitudes;Dixon, 1960;Tukey, 1962).

We statistically compared the group-level human face- and facelike object-selective amplitude differences in each region/hemisphere using permutation tests (2-tailed, 5,000 permutations) and estimated 95% confidence intervals for the differences using a bootstrapping with replacement method.*p*-Values were corrected for multiple comparisons using Benjamini–Hochberg false discovery rate correction (Benjamini & Hochberg, 1995).

### 2.6 Contact localization in the individual anatomy

The exact position of each contact relative to brain anatomy was determined in each participant’s brain by co-registration of the post-operative CT-scan with a T1-weighted MRI of the patient’s head. Anatomical labels of bipolar contacts were determined using the anatomical location of the “active” contact. Bipolar contacts in which the active contact was in the white matter were excluded from amplitude and proportion analyses. To accurately assign an anatomical label to each contact, we used the same topographic parcellation of the VOTC as inJonas et al. (2016), which closely aligns with the parcellation proposed byKim et al. (2000).

### 2.7 Group visualization, proportion, and amplitude analyses in Talairach space

For group analyses and visualization, anatomical MRIs were spatially normalized in order to determine the Talairach (TAL) coordinates of VOTC intracerebral contacts. The cortical surface used to display group contact locations and maps was obtained from segmenting the Collin27 brain from AFNI (Cox, 1996), which is aligned to the TAL space. TAL-transformed coordinates were used to compute maps of the local proportion of category-selective intracerebral contacts across the VOTC. Proportions were computed separately for human face-selective, facelike object-selective contacts, and contacts selective for both (overlap contacts).

Local proportions of contacts were computed in volumes (i.e., “voxels”) of size 15 x 15 x 100 mm (respectively, for the x: left–right, y: posterior–anterior, and z: inferior–superior dimensions) by steps of 3 x 3 x 100 mm over the entire VOTC. A larger voxel size in the z-dimension enabled collapsing across contacts along the inferior–superior dimension. For each voxel, we extracted the following information across all participants in our sample: (1) the number of recorded contacts located within the voxel across all participants; (2) the number of significant human face- and facelike object-selective contacts, as well as overlap contacts. From these values, we computed the proportion of significant human face- and facelike object-selective contacts, and overlap contacts within each voxel, by dividing the number of significant contacts within the voxel by the total number of recorded contacts in that voxel across all participants.

Then, for each voxel and each response pattern (human face-selective, facelike object-selective, overlap) we determined whether the proportions of significant selective contacts were significantly above zero using a percentile bootstrap procedure, as follows: (1) within each voxel, sampling as many contacts as the number of recorded contacts, with replacement; (2) for this bootstrap sample, determining the proportion of significant (human face-selective, facelike object-selective, overlap) contacts and storing this value; (3) repeating steps (1) and (2) 2,000 times to generate a distribution of bootstrap proportions; and (4) estimating the*p*-value as the fraction of bootstrap proportions equal to zero. Following the same principle, we also visualized the variations in the proportions of human face-selective, facelike object-selective, and overlap contacts as a function of the posterior–anterior axis (y-dimension) collapsed across both hemispheres. Proportions were computed along the y-dimension in segments of 12 mm and by steps of 3 mm, collapsing contacts across the x (lateral–medial) and z (inferior–superior) dimensions.

We used the same mapping approach to compute the local category-selective amplitude across the VOTC. For amplitude, we used the winsorized mean human face- and facelike object-selective baseline-subtracted amplitude across contacts within each 15 x 15 x 100 mm voxel over the cortical surface.

### 2.8 Correlation analyses

We performed several correlation analyses to estimate the similarity in the selective response proportion patterns and amplitude in the VOTC regions and in the time course, quantifying the functional correspondence between the two responses (human face- and facelike object-selective). Correlations were computed using paired recording contacts or anatomical regions in each hemisphere (i.e., mean over a set of contacts) as data points. We used a log transformation to normalize the data and then performed a Pearson correlation analysis on the log-transformed data.

We further assessed the strength of the correlations between the amplitudes recorded from the overlap contacts for human faces and facelike objects by comparing them with the maximum expected correlation (MEC) given the noise in the data. The MEC, as described byJacques et al. (2020), is based on an estimation of the test–retest reproducibility of the amplitudes. It was calculated by correlating the measured amplitudes with a simulated noisy measurement, following the approach outlined byKay et al. (2013). More precisely, first, to estimate the noise distribution, we measured the human face- and facelike object-selective amplitude responses independently for each stimulation sequence using the same method as in the main analysis, except for averaging across the sequences first. We then calculated the standard deviation of these amplitudes across the sequences for each overlap contact, separately for the human face and facelike object conditions. Second, to estimate the “true signal” we averaged the amplitudes from these sequences. Using this information, we determined the MEC through Monte Carlo simulations. For each condition separately, in each iteration, we created a simulated noisy set of amplitudes by adding simulated noise—derived by multiplying the noise distribution for each overlap contact with random values from a normal Gaussian distribution to the true signal. We then log-transformed both the measured and simulated amplitudes, calculated the Pearson correlation between these log-transformed values, and recorded the correlations reflecting within-condition reproducibility. After 2,000 simulations, the correlation values for each condition were averaged to obtain the mean reproducibility. The MEC was defined as the smaller of the two reproducibility values (i.e., one for each condition) and was computed for the VOTC as well as individually for each main VOTC region.

### 2.9 Timing of category selectivity

We investigated the time domain responses for recording contacts located in the lateral fusiform gyrus (latFG), where amplitudes were maximal for both conditions. We only included contacts that showed selective responses to both of the condition categories (i.e., the overlap contacts, N = 39 in latFG).

The starting point for the analyses in the time domain was the raw 70 s time series of SEEG recorded for each FPVS sequence. For each recording contact, the time series were processed in the following way: (1) an FFT notch filter (filter width = 0.1 Hz) was applied to remove the general visual response at 6 Hz and 3 additional harmonics (i.e., 6, 12, 18, 24 Hz), and an additional low-pass filter was applied with a cutoff at 30 Hz (4th order Butterworth filter); (2) time series were segmented in 1.17 s epochs centered on the onset of each human face and facelike object in the FPVS sequences; (3) resulting epochs were averaged; and (4) baseline corrected by subtracting the mean amplitude in a [-0.166 to 0 s] time window relative to human face/facelike object stimulus onset. (5) To limit the variability in the response morphology or polarity across recording contacts, the averaged response at each contact was transformed into single polarity variations of amplitude over time by taking the absolute values.

The resulting averaged time domain responses per recording contact were used to determine the response latency of the category-selective responses at each contact. Specifically, the response onset and offset latencies were determined as the points where the EEG signal deviated from the baseline (mean amplitude of the pre-stimulus window, -0.166 to 0 s) by more than ±2.58 times the standard deviation of the baseline (*p*< .01, 2-tailed). Onsets were defined as the first time point exceeding this threshold, sustained for at least 30 ms. Offsets were identified as the first return to within the threshold range, also sustained for a minimum of 30 ms, ensuring transient fluctuations were excluded from the latency estimations. Confidence intervals for the latency estimations were computed using a percentile bootstrap approach as follows: (1) randomly sampling contacts with replacement, (2) averaging the responses from the sampled contacts, (3) repeating steps (1) and (2) 1,000 times, (4) computing a*p*-value for each time point as the fraction of bootstrap samples exceeding the mean amplitude in the pre-stimulus baseline, (5) determining response latency as the time point where*p*< .01 for at least 30 consecutive milliseconds, and recording this latency, (6) repeating steps (1) to (5) 1,000 times to obtain a distribution and confidence interval for the response latencies, before averaging across contacts.

## 3 Results

**Fig. 2.**
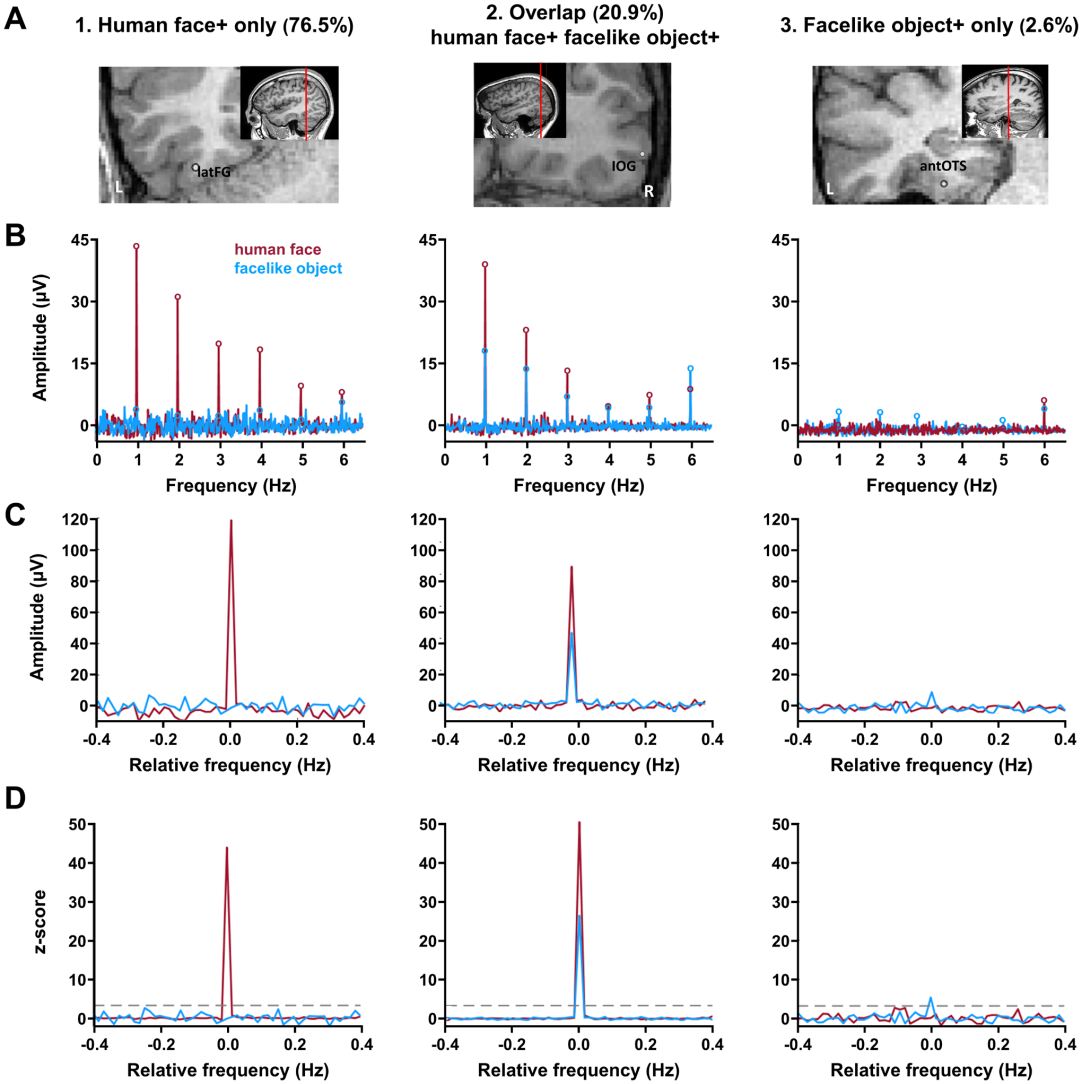
Classification of the three types of category-selective contacts. (A) The anatomical locations of the sample contacts are shown in their respective coronal MRI slices. The letter at the bottom of the images (R/L) refers to the hemispheric side. (B) SEEG frequency domain responses (baseline-corrected FFT amplitudes) recorded at a single recording contact in the human face and facelike object conditions. The human face- and facelike object-selective responses are found at the exact human face and facelike object presentation frequency (1 Hz) and its harmonics. Note the high SNR afforded by the high-frequency resolution (0.0152 Hz). (C) Significant selective responses were determined by first segmenting the FFT spectrum into five segments centered at the frequency of human face and facelike object presentation and its harmonics up to 5 Hz, including surrounding noise within a 0.4 Hz range (i.e., -0.4 to 0.4 Hz centered on 0 Hz). The five segments, containing both the signal and the surrounding noise, were then summed. The 0 mark on the x-axis corresponds to the human face and facelike object presentation frequency. (D) Z-score transformation of the summed FFT spectrum for to statistically identify selective responses. This transformation was computed as the difference between the amplitude at the human face/facelike object presentation frequency bin and the mean amplitude of 48 surrounding bins (25 bins on each side, excluding the 2 bins directly adjacent to the bin of interest, i.e., 48 bins), divided by the standard deviation of amplitudes in the 48 surrounding bins. Contacts with z-scores exceeding 3.1 (*p*< .001; gray dashed line) in the human face condition but not in the facelike object condition were classified as human face+ only contacts (left). Conversely, contacts with z-scores exceeding 3.1 solely for facelike objects and not for human faces were classified as facelike object+ only contacts (right). Finally, contacts that elicited z-scores higher than 3.1 for both conditions were classified as overlap (human face + facelike object+) contacts (middle).

We employed a frequency-tagging visual stimulation paradigm optimized to assess categorical selectivity for human faces and facelike objects (Rekow et al., 2022a,2022b). Human face- and facelike object-selective responses were determined by contrasting, in separate runs, a large set of naturalistic human face or facelike object images to a large set of natural non-face object stimuli depicting various living and non-living object categories. High SNR human face- and facelike object-selective SEEG responses were objectively identified and quantified at the exact human face/facelike object presentation frequency (1 Hz) and harmonics (Fig. 2B). Significant category-selective responses were determined by summing the first five harmonics (i.e., summing 1, 2, 3, 4, and 5 Hz,Fig. 2C) and computing a*z*-score transformation (*z*> 3.1,*p*< .001,Fig. 2D).

### 3.1 Category-selective responses to human faces and facelike objects

**Fig. 3.**
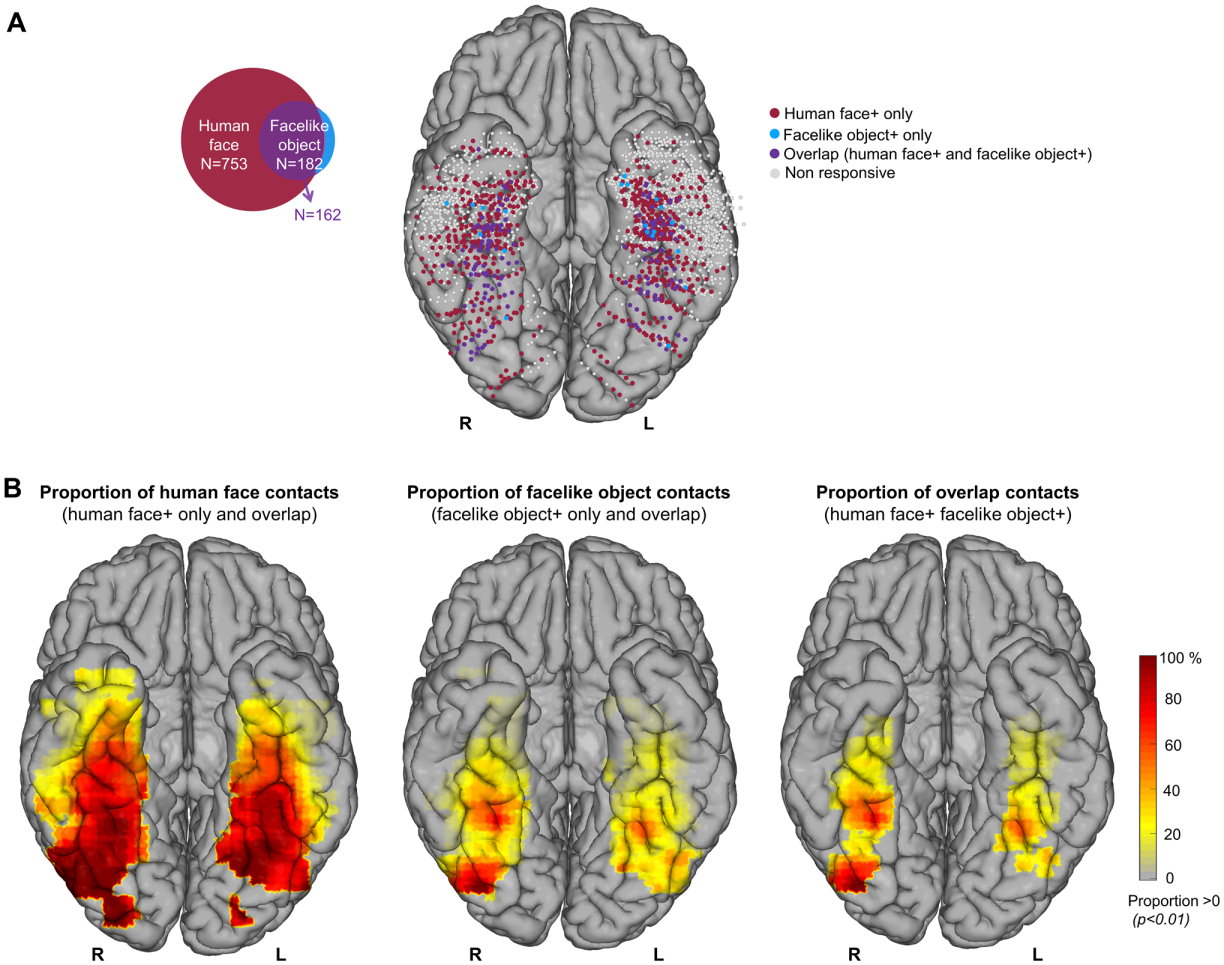
Spatial distribution and proportions of the human face, facelike object, and overlap contacts. (A) Map of all 2,695 VOTC recording contacts across the 44 individual brains displayed in Talairach space using reconstructed cortical surface of Colin27 brain. Each circle represents a single recording contact. Light-gray-filled circles correspond to contacts on which no significant selective responses were recorded. The color-filled contacts correspond to the three types of selective contacts colored as a function of whether human face and/or facelike object activity is significant (z-score > 3.1,*p*< .001). Counts of the three types of selective contacts are present on the Venn diagram (left). (B) Maps of local proportion of selective contacts relative to recorded contact across VOTC for each contact type. The local proportions are computed in 15 x 15 mm voxels for x (medio-lateral) and y (postero-anterior) Talairach dimensions, respectively, collapsing contacts across z-dimension (inferior–superior) for visualization. Only voxels with a proportion of selective contacts significantly above zero (*p*< .01, percentile bootstrap) are displayed.

At this statistical threshold, we identified 773 contacts with category-selective responses (a differential response for human faces and/or facelike objects as compared with non-face objects) among the recorded 2,695 bipolar contacts located in the VOTC of 44 participants (i.e., 29% of recorded contacts). Among these category-selective responses, 76.5% were selective for human faces only (591 out of 773, 44 patients), 20.9% were selective for both human faces and facelike objects (162 out of 773, 38 patients), and the remaining 2.6% were selective for facelike objects only (20 out of 773, 13 patients;Fig. 3A). This generated a strong asymmetry between the 2 categories, both in the total number of selective contacts (~4 times more human face contacts, 182 vs*.*753) and in the percentage of overlap contacts between the categories. Specifically, most facelike object-selective contacts (89%, 162 out of 182) showed selective activity also for the human faces, while only 21.5% (162 out of 753) of the human face-selective contacts also showed selective responses to facelike objects. In contrast, whether sequences contained human faces or facelike objects did not impact the total number of general visual response-selective contacts (884 vs*.*853 out of 2,695 for human faces and facelike objects, respectively) or the percentage of overlapping contacts (75% vs*.*77.7%, 663 in both).

The few remaining contacts responding only to facelike objects (non-overlapping; 2.6%, 20 out of 773) were scattered across the VOTC (Fig. 3A). In contrast, the 591 human face+ only contacts exhibit a distribution pattern similar to the 162 contacts that respond to both human faces and facelike objects (i.e., overlap contacts), as confirmed by a comparison of the weighted center of mass coordinates for the x (medio-lateral) and y (postero-anterior) Talairach dimensions. These comparisons, calculated for overlap and human face+ only contacts, do not reveal any significant differences in either hemisphere; RH (mean_xdiff_, [95% confidence interval] = 1.10 mm, [-1.54, 3.74], mean_ydiff_= 1.96 mm, [-4.65, 9.01]) and LH (mean_xdiff_= 0.39 mm, [-1.98, 2.69], mean_ydiff_= 5.47 mm, [-3.75, 14.48]).

### 3.2 Spatial overlap between human face and facelike object activity in the VOTC

To compare and visualize the spatial organization of the human face, facelike object, and overlap contacts at the group level, we computed the local proportion of category-selective contacts (relative to the total number of recorded contacts) and projected them on the cortical surface. Each category-selective contact was located and labeled according to the participant’s individual anatomy, using a typical topographic parcellation of the VOTC (as described inJonas et al., 2016; seeTable 1for the number of significant contacts in each anatomical region).

**Table 1.** Number of selective contacts and corresponding number of participants (in parentheses) in each anatomical region.

	LH			RH		
Region	Human faces	Facelike objects	Overlap	Human faces	Facelike objects	Overlap
**VMO (OCC)**	19 (5)	3 (2)	3 (2)	18 (5)	3 (1)	2 (1)
**IOG (OCC)**	18 (6)	6 (4)	5 (3)	32 (6)	12 (4)	12 (4)
**medFG (PTL)**	23 (10)	5 (4)	5 (4)	25 (11)	10 (6)	10 (6)
**latFG (PTL)**	59 (17)	21 (10)	20 (10)	39 (15)	19 (10)	19 (10)
**MTG/ITG (PTL)**	19 (10)	0 (0)	0 (0)	31 (11)	4 (4)	4 (4)
**antCOS (ATL)**	81 (29)	21 (9)	18 (9)	44 (22)	10 (9)	7 (7)
**antFG (ATL)**	17 (12)	4 (3)	4 (3)	17 (10)	7 (6)	7 (6)
**antOTS (ATL)**	73 (26)	19 (12)	15 (8)	86 (27)	26 (15)	24 (13)
**antMTG/ITG (ATL)**	34 (19)	4 (3)	3 (2)	40 (13)	2 (2)	1 (1)
**TP (ATL)**	7 (5)	0 (0)	0 (0)	13 (5)	1 (1)	1 (1)
**AMG (MTL)**	11 (8)	4 (3)	1 (1)	16 (9)	1 (1)	1 (1)
**HIP (MTL)**	17 (11)	0 (0)	0 (0)	11 (7)	0 (0)	0 (0)
**Total**	378	87	74	372	95	88

Note. Each region is followed by its broader anatomical subdivision in parentheses. Acronyms: VMO: ventro-medial occipital cortex; IOG: inferior occipital gyrus; medFG: medial fusiform gyrus and collateral sulcus; latFG: lateral FG and occipito-temporal sulcus; MTG/ITG: the inferior and middle temporal gyri; antPHG: anterior PHG; antCOS: anterior collateral sulcus; antOTS: anterior OTS; antFG: anterior FG; antMTG/ITG: anterior MTG and ITG; AMG: amygdala; HIP: hippocampus; TP: temporal pole; OCC: occipital lobe; PTL: posterior temporal lobe; ATL: anterior temporal lobe; MTL: medial temporal lobe. Several regions were not considered due to having too few contacts (i.e., parahippocampal gyrus- PHG and anterior PHG).

The local proportions of category-selective contacts reveal that selective responses for both human faces and, to a slightly lesser extent, facelike objects are widely distributed throughout the VOTC. The maps reveal a notable concentration along the stretch of cortex extending from the inferior occipital gyrus (IOG) through the fusiform gyrus (FG) to the anterior fusiform gyrus (antFG) for both the human face and facelike object contacts (Fig. 3A, B). Notably, this distribution remains similar for the overlap between the contacts selective for both human faces and facelike objects, which is only different from facelike object contacts by 20 contacts. The highest proportion of overlap contacts relative to the total number of recorded contacts in each anatomical region is found in the right latFG (40.4%) followed by right antFG (38.9%) and right IOG (36.4%), while the lowest proportions were recorded in the anterior middle temporal gyrus/inferior temporal gyrus (antMTG/ITG; 0.66%) and temporal pole (TP; 0.77%). However, the human face+ only contacts are predominantly located in the lateral aspects of the VOTC in posterior temporal lobe (PTL) and anterior temporal lobe (ATL), including the middle temporal gyrus/inferior temporal gyrus (MTG/ITG), anterior occipito-temporal sulcus (antOTS), and antMTG, as well as in more anterior regions such as TP). Additionally, they are found in sub-cortical structures including the amygdala (AMG) and hippocampus (HIP). As discussed above, the few facelike object+ only contacts (N = 20 out of 182) are scattered without a particular clustering. Local proportion analysis did not reveal any VOTC region where the proportion of facelike object only+ contacts relative to total recorded contacts reached significance (Supplementary Fig. S1A).

Overall, there is a higher proportion of human face contacts compared with facelike object contacts (27% vs*.*6.6%,*p*< .001, 2-tailed permutation test) and overlap (27% vs*.*5.9%,*p*< .001), but no difference between facelike object contacts and the overlap (6.6% vs*.*5.9%,*p*= .3). The VOTC maps also reveal that the proportion of selective contacts relative to the total recorded contacts is greater in the right hemisphere than in the left for human face-selective contacts (RH = 33.8% vs. LH = 22.9%,*p*< .001, 2-tailed permutation test), but also for facelike objects (RH = 8.6% vs. LH = 5.2%,*p*< .001) and for the overlap (RH = 8% vs. LH = 4.5%,*p*< .001).

Next, we examined whether the spatial overlap implies a functional association by analyzing the spatial patterns of the response amplitudes and calculated the correlation between the selective amplitudes in the anatomical regions that contained at least one overlap contact (seeSection 2). Similarly, we investigated a functional association between the selective response amplitudes in the overlap contacts and the human face+ only and facelike object+ only contacts with the respective selective responses despite the spatial distinction we observed.

**Fig. 4.**
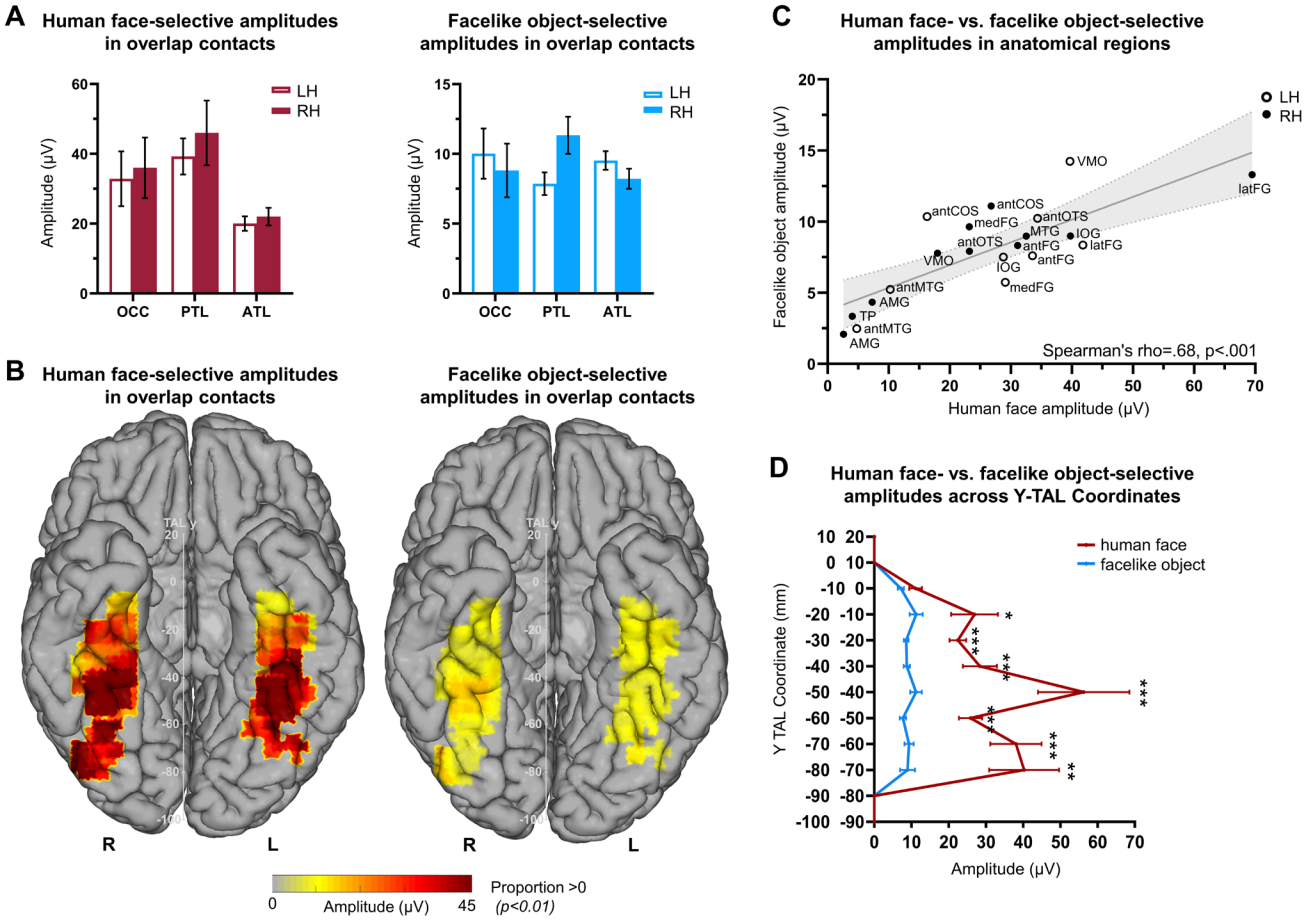
Selective amplitude quantification for human face- and facelike object-selective responses recorded in the overlap contacts across VOTC. (A) Human face-selective amplitudes in overlap contacts (left) and facelike object-selective amplitudes in overlap contacts (right). Amplitudes are quantified as the mean amplitude recorded in each individually labeled main anatomical region and hemisphere. (B) VOTC maps of amplitudes in the overlap contacts for human faces and facelike objects. Amplitudes are quantified as the mean of the amplitudes across overlap contacts within 15 x 15 mm voxels. Only voxels with a proportion of overlap contacts significantly above zero (*p*< .01, percentile bootstrap) are displayed. (C) Scatter plot presenting the similarity in the patterns of mean selective amplitudes recorded from the overlap contacts for human faces and facelike objects in the anatomical regions. Each data point shows the recorded selective mean amplitudes for the two conditions in each anatomical region and hemisphere. The shaded area shows the 95% confidence interval of the linear regression line. (D) Selective amplitudes recorded in overlap contacts as a function of the position of contact along the y Talairach axis (posterior–anterior) computed by collapsing contacts over both hemispheres.

We first investigated the winsorized mean amplitudes across category-selective contacts considering the entire VOTC and subsequently within each individually defined anatomical main region and hemisphere (Fig. 4A, C). We excluded regions with either none or too few overlap contacts as we could not reliably measure and compare amplitudes between the two hemispheres (i.e., TP, MTL).

We observed that the category-selective responses recorded in the overlap contacts showed higher amplitudes for both categories compared with the selective responses recorded from the human face+ only and facelike object+ only contacts. Specifically, human face-selective amplitudes in overlap contacts (mean = 29 μV) were significantly greater than those in human face+ only contacts (mean = 15.3 μV, mean difference = 13.7 μV, 95% CI [5.6, 40.2],*p*< .001). Similarly, facelike object-selective amplitudes in overlap contacts (mean = 8.8 μV) were significantly higher than those in facelike object+ only contacts (mean = 6.2 μV, mean difference = 2.6 μV, 95% CI [2.2, 5.5],*p*< .001).

In the overlap contacts, we found higher selective amplitudes across the VOTC for human faces compared with facelike objects. In particular, the facelike object-selective amplitudes (mean = 9.8 μV) are 27% of the human face-selective amplitudes (mean = 35.8 μV, mean_diff_= 20.2 μV, 95% CI [17.07, 23.49],*p*< .001,Fig. 4B, D). The amplitudes remain lower for facelike objects compared with human faces across the y Talairach axis (posterior–anterior), starting at*y*= -80 to -70, and lasting till*y*= -20 to -10. In contrast, the amplitudes of general visual response-selective contacts were not modulated by the stimulus category presented at 1 Hz (mean_diff_= 0.16 μV, 95% CI [-0.23, 0.55],*p*= .39,Supplementary Fig. S3B).

Despite this lower amplitude overall for the facelike object compared with the human face-selective response, amplitude variations across the anatomical regions present similarities between the two categories. In the overlap contacts, the largest selective amplitudes for human faces and facelike objects were found in the right PTL followed by OCC and then ATL. In accordance with this finding, the VOTC maps in Talairach space also indicated local amplitude peaks observed in the right latFG and left VMO in both conditions (Fig. 4B). In this region with highest amplitude peaks, the right latFG, the weighted center of mass coordinates for the overlapping contacts (N = 39) were centered at x = 37 mm, y = −45 mm for human faces and at x = 39 mm, y = −44 mm for facelike objects. Moreover, consistent with previous SEEG investigations on the neural basis of face categorization (e.g.,Hagen et al., 2020;Jonas et al., 2016), responses were larger in the right PTL (specifically, the latFG with mean difference = 27.7 μV, 95% CI = [-8.73, 68.56]) compared with the left. However, this difference did not reach significance in human face response (mean difference = 7.27 μV, 95% CI = [-12.30, 28.39],*p*= .77). Similarly, although we observed a trend toward significance in facelike object responses, it was no longer significant following FDR correction for multiple comparisons (mean difference = 3.47 μV, 95% CI = [0.54, 6.57], uncorrected*p*= .044, FDR corrected*p*= .12). Despite the hemispheric advantage we reported previously in the proportion of selective overlap contacts, we did not find a clear hemispheric advantage in amplitude across VOTC or in any of the other main regions for either condition (all*p*’s > .18,Fig. 4A).

Lastly, the similarities observed between the amplitudes of the conditions are reflected in the strong linear relationship when evaluating responses of the overlap contacts across the anatomical regions of VOTC (Spearman’s*rho*= .68, 95% CI [.33, .87],*p*< .001 calculated using the mean amplitudes from contacts recorded in the 10 anatomical regions in each hemisphere,Fig. 4C).

### 3.3 Human face- and facelike object-selective amplitudes are functionally associated

We reported a significant spatial overlap between the contacts selectively responding to human faces and those responding to facelike objects (compared with non-face objects), with overlap contacts comprising 89% of the total number of facelike object contacts. We also showed a correlation in the patterns of selective amplitudes for human faces and facelike objects at the anatomical region level (mean amplitudes). While informative, neither the spatial overlap nor the correlation at the anatomical region level signify a functional correspondence between the two category-selective responses. Given that we record local field potentials (i.e., the averaged activity of millions of neurons), the recorded selective responses could arise from distinct but nearby populations of neurons and yet appear as overlap due to spatial proximity (as previously observed with house- vs*.*face-selective responses byHagen et al., 2020). Therefore, if there is a functional association and the responses originate from similar populations of neurons, we expect similar response profiles, resulting in a high correlation. Alternatively, if the response profiles are dissociated resulting in low correlation, it would suggest that the same contacts captured activity from largely disconnected neural populations.

For this reason, we explored the functional relationship between the human face and facelike object category-selective responses by quantifying the correlation (Pearson’s*r*) between the log-transformed amplitude responses across the overlap contacts. We used single recording contacts as data points, correlating the responses recorded from the same contact for the two conditions. This revealed a strong relationship across the responses recorded throughout the VOTC (*r*= .66, 95% CI [.58, .86],*p*< .001), indicating that the selective responses to human faces and facelike objects were also strongly functionally associated.

**Fig. 5.**
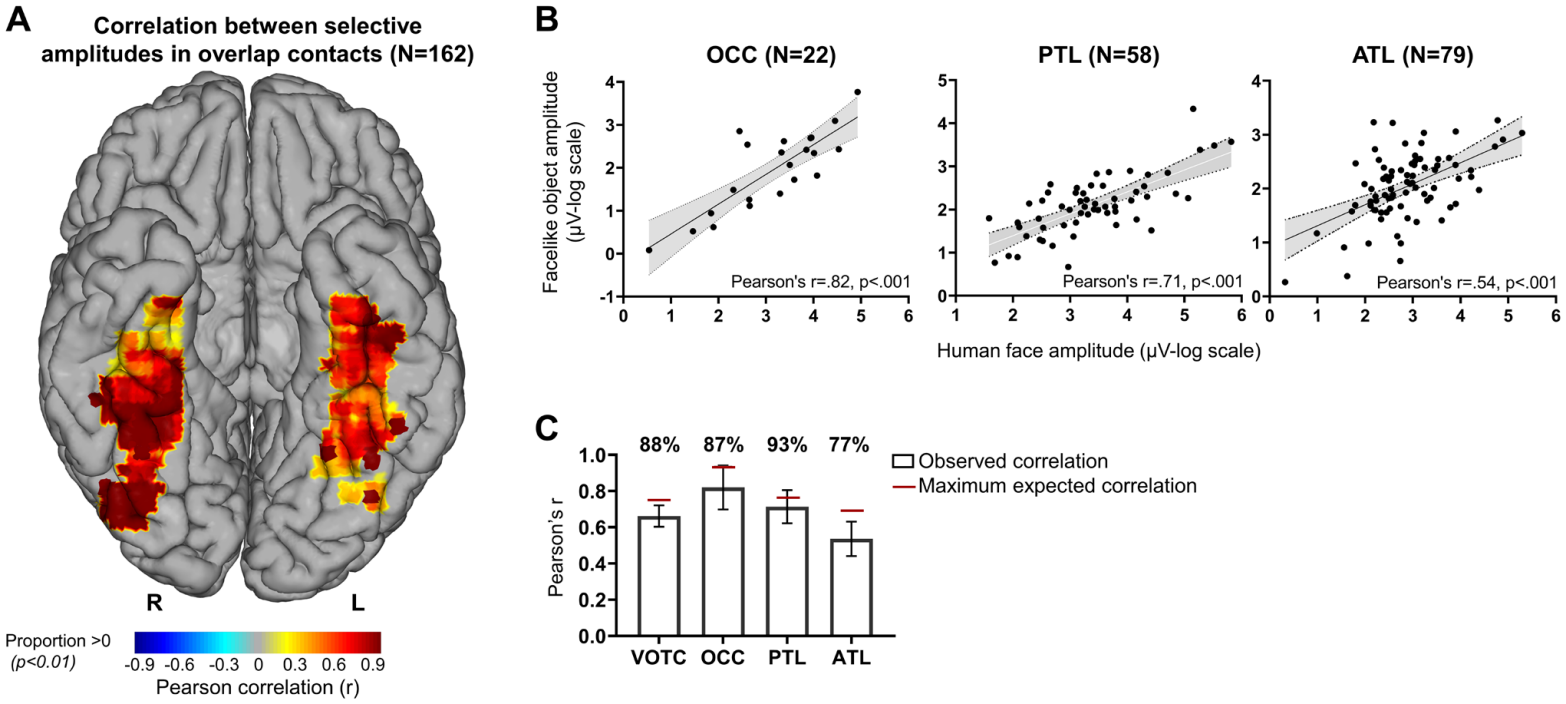
Functional association between human face and facelike object responses in the overlap contacts. (A) VOTC map of Pearson’s*r*calculations between the log-transformed selective responses to human face and facelike object. Correlations were computed using contacts located in 15 x 15 mm voxels. Only voxels with a proportion of overlap contacts significantly above zero (*p*< .01, percentile bootstrap) are displayed. (B) Scatter plots presenting a linear relationship between the log-transformed amplitude responses for human faces and facelike objects in the overlap contacts in the three anatomical main regions (OCC, PTL, ATL). (C) Pearson’s*r*values for the whole VOTC and each main region (as shown in the bars) presented with the maximum expected correlation (MEC, as shown by red lines) considering the noise in each dataset. The percentages above each bar represent the ratio of actual correlation to the MEC, indicating the percentage of the maximum expected correlation achieved in each region.

We investigated the correlations separately for each main region in the VOTC, and additionally, we mapped the Pearson’s*r*correlation values calculated in 15 x 15 mm voxels to investigate the variations in correlation across the VOTC at a finer scale (Fig. 5A). We found significant correlations in each of the main regions. The highest correlation was found for OCC (*r*= .82, 95% CI [.61, .92],*p*< .001) followed by PTL (*r*= .71, 95% CI [.56, .82],*p*< .001) and ATL (*r*= .54, 95% CI [.36, .68],*p*< .001,Fig. 5B). In line with those, the highest significant correlations were in the IOG (Pearson’s*r*= .87 [.52, .98],*p*< .001), the latFG (*r*= .69, 95% CI [.59, .93],*p*< .001), and the antFG (*r*= .75, 95% CI [.35, .94],*p*< .001). It is noteworthy that despite the spatial overlap between the selective contacts in human face and facelike object conditions within the medial part of the fusiform gyrus (medFG), the amplitudes of the two conditions were not significantly correlated in this region (*r*= .38, 95% CI [-.02, .69],*p*= .24), in contrast with the latFG.

Understanding the strength of the relationship between human face and facelike object responses also depends on the maximum correlation that can be expected given the data’s noise levels. To account for this, we computed an estimation of the maximum expected correlation (MEC) in our datasets which reflects test–retest reliability. The noise considered for MEC calculation was determined by the standard deviation in human face- and facelike object-selective response amplitude across the FPVS sequences. The MEC at the whole level of VOTC was*r*= .75, 95% CI [.58, .86], which meant that the correlation between the human face and facelike object responses reached 88% of the MEC (.66/.75) in this dataset. In the main regions of VOTC, the correlations between the human face and facelike object responses reached 87% (OCC, .82/.94), 93% (PTL, .71/.77), and 77% (ATL, .54/.69) of the MEC.

Moreover, we investigated the relationship between human face and facelike object responses in the largest group of category-selective contacts (i.e., human face+ only contacts, n = 591). We hypothesized that, given the absence of significant facelike object-selective responses in these contacts, a lack of correlation would further indicate that these regions are specifically involved in human face processing. Conversely, a significant correlation would suggest that, although involved in facelike object processing, these regions were not fully engaged by facelike stimuli as they were by human faces. Interestingly, despite the lack of selectivity for facelike objects, we found a significant correlation between responses to the two categories across the whole VOTC (Pearson’s*r*= .37, 95% CI [.30, .44],*p*< .001), and in the main regions of VOTC (OCC,*r*= .35, 95% CI [.12, .55],*p*= .01; PTL,*r*= .38, 95% CI [.23, .51],*p*< .001; ATL,*r*= .39, 95% CI [.30, .49],*p*< .001,Supplementary Fig. S2A). The correlations between the human face responses recorded from the human face+ only contacts and overlap contacts reached 75% (OCC, .35/.47), 88% (PTL, .38/.43), and 98% (ATL, .39/.40) of the MEC (Supplementary Fig. S2B).

### 3.4 Face- and facelike object-selective responses show concurrent time courses

We explored the time course relationship between the selective responses to human faces and facelike objects, hypothesizing that a correspondence between the timing of the selective responses would further suggest the existence of a functional overlap between human face- and facelike object-selective responses in the VOTC. To investigate the time courses, we focused on the latFG, the anatomical sub-region with the largest amplitudes, and a high number of selective overlap contacts for the two categories (N = 39).

**Fig. 6.**
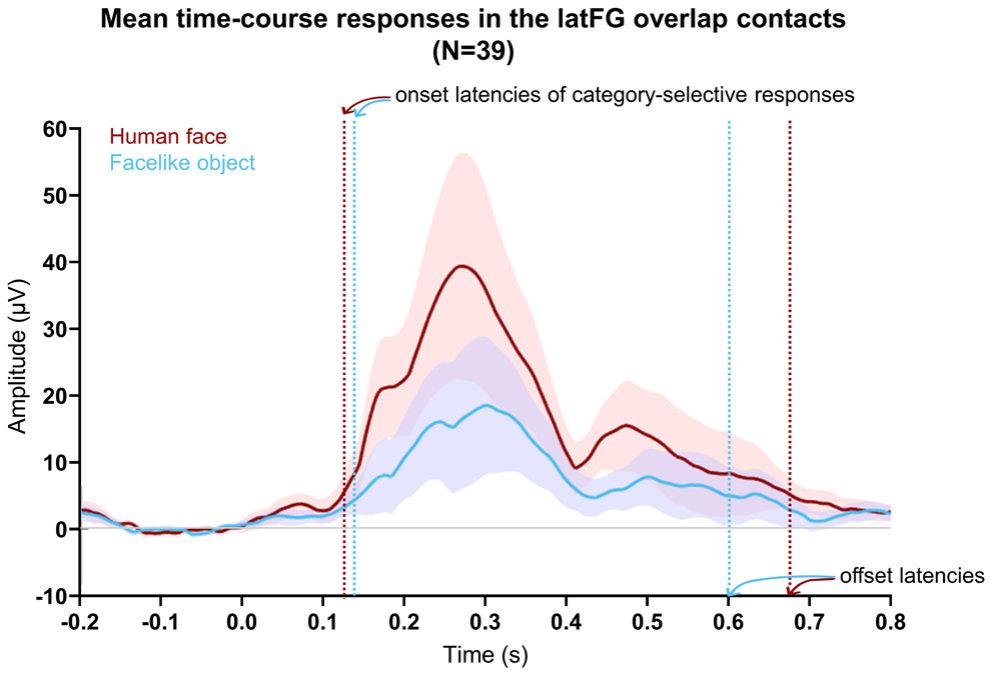
Mean time domain responses for human faces and facelike objects on overlap contacts located in the latFG (N = 39). FPVS sequences were segmented relative to each human face and facelike object onset (i.e., every 1 s). The signal related to the general visual response at 6 Hz and harmonics was selectively filtered out. The time domain responses were transformed to absolute values to mitigate the impact of variation in response morphology and polarity across recording contacts before averaging. Vertical lines show estimated onset and offset latencies for human face and facelike object-selective responses. The shaded areas show the 95% CI across contacts at each time point.

The averaged time course of the overlap contacts in the latFG showed that both selective responses started to deviate from baseline at about the same latency (onset latency for human face = 126 ms, 95% CI [107, 140 ms], facelike object = 136 ms, 95% CI [116, 190 ms];Fig. 6). While the facelike object response (offset latency = 603 ms, 95% CI [393, 690 ms]) returned to baseline slightly earlier than the human face response (673 ms, 95% CI [524, 690 ms]), the time courses across time [0 to 700 ms] were highly correlated (*r*= .96, 95% CI [.95, .97],*p*< .001).

## 4 Discussion

Our comprehensive intracerebral recording of the selectivity for human faces and facelike objects in the human VOTC reveals four key findings. First, regions that selectively respond to facelike objects, compared with non-face objects, are spatially distributed yet clustered in the VOTC within a narrow strip of cortex extending from the bilateral IOG and latFG to regions of the ATL. Second, the distributed facelike object-selective responses largely overlap with the ventral human face categorization network. Specifically, the vast majority of contacts showing facelike object-selective activity also exhibit human face-selective activity, with the highest overlaps observed in critical face network sites. Third, amplitudes for facelike objects were substantially reduced. Fourth, face and facelike neural responses are robustly correlated across regions, recording contacts and time courses.

### 4.1 Widely distributed facelike-selective responses in the VOTC

The wide spatial distribution of face-selective neural activity across the VOTC compared with non-face objects has been previously described (Hagen et al., 2020;Jacques et al., 2022;Jonas et al., 2016). Our study provides an original contribution by showing that selective responses discriminating facelike objects from non-face objects are also widely distributed. Specifically, we demonstrate that visual processing of facelike objects is achieved by widely distributed populations of neurons clustering primarily within a certain stretch of cortex in both hemispheres. This includes the bilateral IOG, latFG, and extends to more anterior VOTC regions (antCOS, antFG, and antOTS) that are difficult to investigate with fMRI due to significant signal drop out from magnetic susceptibility artifacts (Rossion et al., 2024;Wandell, 2011).

Previous fMRI studies have identified facelike object-selective responses, compared with non-face objects in the IOG (OFA;Decramer et al., 2021;Wardle et al., 2020) and latFG (FFA;Wardle et al., 2017,^2020^), as well as in some bridging regions between the IOG and latFG. These areas all fall within the stretch of cortex we have identified. Our study extends these findings by identifying additional regions that exhibit facelike object-selective responses, including the medFG, MTG/ITG, and vATL (antCOS, antFG, and antOTS).

### 4.2 Facelike object-selective responses highly overlap with the ventral human face categorization network

Previous studies have provided evidence for common brain regions exhibiting selective responses to human faces and face pareidolia across different imaging modalities. These include spatial overlaps in EEG scalp topography (Caharel et al., 2013;Churches et al., 2009;Proverbio & Galli, 2016;Rekow et al., 2022a,2022b), source-localized MEG activity (Hadjikhani et al., 2009;Romagnano et al., 2024;Wardle et al., 2020), within face-selective VOTC regions (i.e., FFA, OFA) in fMRI (Akdeniz et al., 2018;Boccia et al., 2015;Wardle et al., 2017,^2020^), and in the OFA of a single patient with single cell recordings (Decramer et al., 2021).

In line with these studies while providing a much more comprehensive investigation, we observed that facelike object-selective responses highly overlap with human face-selective responses. Specifically, the vast majority of contacts showing facelike object-selective activity (162 out of 182, 89%) also exhibited selectivity to human faces. The few remaining contacts (20 out of 182, 11%) that responded exclusively to facelike objects were widely scattered, exhibited low amplitudes, and did not cluster in any specific anatomical region. Although these exclusively facelike object-selective contacts appeared to be primarily located in the anterior regions, this is likely due to the denser sampling in the ATL region, as shown inFigure 3A. Additionally, a VOTC map depicting the proportion of exclusively facelike object-selective contacts relative to the recorded contacts (Supplementary Fig. S1A) reveals no VOTC location with a proportion of facelike object+ only contacts significantly above zero. These findings suggest that within the VOTC regions examined here, there is no distinct brain area dedicated to facelike object processing without also being selective for human faces or non-face objects.

The current findings highlight a significant overlap of recording contacts within key regions of the face processing network, with the highest proportions observed in the right latFG (40.4%), then the right antFG (38.9%) and the right IOG (36.4%). These regions are well established as critical nodes of a highly interconnected face-selective network (Jonas & Rossion, 2021;Angelini et al., 2024). Notably, the right latFG consistently shows robust face-selective fMRI activity, that is, the FFA (Chen et al., 2022;Grill-Spector et al., 2017;Kanwisher et al., 1997), with damage of temporary inactivation of this region being frequently associated with impairments in face identity recognition (Barton et al., 2002;Cohen et al., 2019;Volfart et al., 2023). The right IOG is also consistently associated with high face-selective fMRI activity (occipital face area-OFA, e.g.,Gauthier et al., 2000;Haxby et al., 2000) and also appears crucial for face identity recognition, as indicated both by lesion studies (Bouvier & Engel, 2006;Rossion et al., 2003) and direct electrical stimulation (Jonas et al., 2012,^2014^).

Both the latFG and IOG have been implicated in face pareidolia, as evidenced by fMRI (Akdeniz et al., 2018;Boccia et al., 2015;Wardle et al., 2017,^2020^) and single-cell recordings (Decramer et al., 2021). In contrast, the contribution of the anterior fusiform gyrus (antFG) in face pareidolia has remained largely unexplored due to its location within the significant magnetic susceptibility artifact zone in fMRI. This region is thought to be involved in processing faces increasingly independently of the visual context and in which face information is shared with other modality-specific regions in order to build semantic associations (Rossion et al., 2024). Notably, transient inactivation of the right antFG by intracerebral electrical stimulation also impairs human face identity recognition (Jonas et al., 2015;Volfart et al., 2022). More generally, the involvement of ATL regions (antCOS, antFG, antOTS) in rapid automatic visual perception of (unfamiliar) human faces challenges the view that areas anterior to the middle fusiform gyrus (midFG) should be excluded from the cortical face network because they would be associated with multimodal semantic memory rather than visual processing (Grill-Spector et al., 2017). Building on this, the relative involvement of vATL regions in the processing of facelike stimuli as revealed here is notable. Even though these stimuli may not be as strongly tied to semantic associations as human faces (e.g., identity, social cues, emotions, age, and gender), ATL regions were as responsive as the rest of the facelike object-selective VOTC network, in fact even relatively more responsive. Thus, our findings indicate that similar to human faces (Jonas et al., 2015;Rossion et al., 2003,^2024^;Volfart et al., 2022), the anterior regions of the VOTC play a role in face pareidolia, supporting their broader role in visual perception. This underscores the necessity to consider these brain areas in future research that aims to fully understand how the ventral human face categorization network supports face perception-related phenomena.

### 4.3 Neural divergence between human face and facelike object processing

In line with previous studies (Caharel et al., 2013;Decramer et al., 2021;Rekow et al., 2022a,2022b;Wardle et al., 2020), we observed a substantially diminished selective response to facelike objects compared with human faces. This was evident in both the number and spread of significant contacts and the amplitude of their responses. While both facelike objects and human faces activated the same face-selective regions along the VOTC, facelike object processing engaged only a subset of the ventral network activated by the human faces. This selective engagement may contribute to the diminished scalp response observed for face pareidolia compared with human faces (Caharel et al., 2013;Rekow et al., 2022a,2022b). As previously discussed, the subset of the face network involved in processing facelike objects was primarily concentrated in critical face-processing regions, whereas the MTG, antMTG, TP, and hippocampus showed the lowest overlap between the category-selective contacts across both hemispheres.

This latter observation may reflect a distinction between the cognitiveassociativeand emotional depth involved in recognizing real human faces versus facelike objects. The listed regions, particularly the MTG and TP, are known to be engaged in the complex semantic and social processing that human faces evoke, including familiar identity recognition, memory retrieval, and linking of familiar faces with abstract concepts related to the person (Deen et al., 2024;Kirwan & Stark, 2004;Kriegeskorte et al., 2007;Olson et al., 2007;Rajimehr et al., 2009). The hippocampus is similarly implicated in associating faces with specific (cortical) memories and emotional contexts (Fenker et al., 2005;Hayes et al., 2010;Leveroni et al., 2000).

In contrast, face pareidolia likely activates a more limited set of perceptual processes, insufficient to fully engage these regions, which are more responsive to the nuanced and context-rich nature of the genuine human face recognition (Haxby et al., 2000;Kanwisher & Barton, 2011). Even though emotion and expression detection (Alais et al., 2021;Wardle et al., 2022), gender perception (Lipp & Taubert, 2024;Wardle et al., 2022), and even instances of identity recognition (e.g., Jesus on toast,Healy, 2009) are also present in face pareidolia experiences, this highlights the more superficial processing involved in recognizing facelike objects, which may not invoke the deeper associative and emotional networks to the same extent as human faces.

Note, however, that even in these regions showing significant responses to human faces but not facelike objects, such as the lateral portions of the VOTC in the PTL and ATL (i.e., MTG/ITG, antOTS, and antMTG), as well as in more anterior areas like the TP, we observed significant correlations between responses to the two categories, indicating functional association in these regions.

Importantly, the activation of only a subset of the face-selective network is not sufficient to explain the diminished average response on its own. Indeed, even when considering only the overlap contacts—those showing selective responses to both human faces and facelike objects—the amplitude of the response to facelike objects was significantly reduced, registering at just 27% of that observed for human faces. This reduction in amplitude may reflect a less consistent or less robust activation of the face-selective network when processing facelike object stimuli. This diminished response could be tied to the variability in the features of these stimuli, as well as the subjective perception of them as faces. To give an example, faces share a range of diagnostic color information under natural lighting conditions, which facilitates fast and efficient face categorization (Liu-Shuang et al., 2022). The absence of typical biological facial features in facelike objects, including diagnostic color information, may lead to lower overall similarity among these objects compared with human faces, and a higher similarity to non-face objects (Wardle et al., 2020). This variability and lack of informative color cues in facelike objects may contribute to reduced neural activation.

Alternatively, the two stimuli might elicit comparable responses within the subset of regions selective for both human faces and facelike objects, but this might only occur when facelike objects evoke perceptual awareness of a face. Given the subjective variability in the experience of face pareidolia, even with the use of a pre-selected list of highly facelike images (i.e., seeRekow et al. 2022a), an inconsistent perception of facelike objects as faces might decrease the averaged response to facelike objects due to the less frequent occurrence of facelike perceptions (seeRetter et al., 2020). To circumvent this limitation of the present study, and address this issue in future studies, one would have to measure explicit perception of faces in such facelike stimuli trial-by-trial in independent stimulation sequences or post-recording sessions, averaging trials or sessions only associated with explicit perception of faces (i.e., pareidolia).

While our findings corroborate the reduced amplitudes observed byRekow et al. (2022a), we did not observe the increased right hemispheric lateralization for facelike stimuli reported in that EEG study. In fact, although a right hemispheric advantage was present in the proportion of significant contacts, response magnitudes for facelike objects and human faces did not show significant hemispheric differences in the present study. This observation is somewhat surprising given the consistent right hemispheric advantage observed with this paradigm measuring face categorization in scalp EEG (e.g.,Rossion et al., 2015;Retter & Rossion, 2016), fMRI (Gao et al., 2018) and, most importantly, intracerebral recordings (Jonas et al., 2016;Hagen et al., 2020,^2021^;Jacques et al., 2022). However, seeHauk et al. (2021)for only a numerical but non-significant right hemisphere advantage in MEG.

While one may be tempted to attribute the lack of right hemispheric advantage to a putative atypical functional lateralization of the epileptic brain (Berl et al., 2014;Powell et al., 2007), this is unlikely given previous evidence of right lateralization with this very paradigm in the same patient population (Jonas et al., 2016;Hagen et al., 2020,^2021^;Jacques et al., 2022; see alsoJacques et al., 2020for a substantially larger right hemispheric lateralization in face individuation). Instead, a key factor might be the referencing approach. Here, contrary to previous studies, we used a local re-referencing approach (bipolar reference), where the signal at each recording contact was calculated by subtracting the signal measured at the immediately adjacent contact positioned more medially on the same SEEG electrode array (Li et al., 2018). This approach has many advantages, such as mitigating dependencies between neighboring contacts, but it also might be detrimental to the investigation of hemispheric advantages. Specifically, if sources are stronger in the right hemisphere, but responses share some commonality between adjacent contacts, the bipolar referencing by subtracting the signal measured at the immediately adjacent contact could reduce sensitivity to hemispheric differences. This limitation has in fact been recently revealed by a related piece of work (Hagen et al., 2025), where the findings advocate for the strengths of bipolar referencing while noting its constraints for assessing hemispheric lateralization.

### 4.4 Human face- and facelike object-selective responses in the VOTC are functionally associated

We found that 89% of facelike object-selective contacts spatially overlap with face-selective regions. While such a spatial overlap may result from local field potentials reflecting the aggregated activity of distinct but nearby neuronal populations (Hagen et al., 2020), responses recorded from the same recording contacts for human faces and facelike objects show high amplitude correlations across the VOTC and in each of its main regions, supporting their functional association. Correlation coefficients approached the maximum expected ceilings, considering noise, reaching 88% of the maximum expected correlation in the VOTC, 87% in the occipital cortex (OCC), 93% in the posterior temporal lobe (PTL), and 77% in the anterior temporal lobe (ATL).

Furthermore, the timing of selective responses also supports the notion of functional overlap. In accordance with previous findings (Caharel et al., 2013;Churches et al., 2009;Proverbio & Galli, 2016), we observed early temporal correspondences between human face and facelike object responses in the latFG, which challenges the view that facelike objects are re-interpreted as faces after an initial categorization as a non-face object. Instead, these findings suggest that recognition of facelike objects is more accurately attributed to their sensory inputs matching memories of faces in the (visual) association cortex, similar to the recognition of human faces. This matching process must be informed by a broad range of visuo-semantic experiences, allowing for a generalized and abstract recognition rather than a strictly localized analysis of physical features. Building on the temporal correspondence observed here in the latFG, future research examining the timing of human face- and facelike object-selective responses across the whole VOTC would help better understand the extent of this correspondence.

### 4.5 Conclusions

Using intracerebral recordings from a large group of individual brains, we show that selective activity for facelike objects occurs across all key regions of the human cortical face network, including the previously unexplored anterior temporal lobe (ATL). While category-selective responses to facelike objects were reduced in amplitude compared with human faces, 89% of facelike object-selective contacts overlapped with face-selective contacts, and both stimuli exhibited highly correlated neural signals with similar onset times. These findings challenge the notion that facelike objects are interpreted as faces through feedback from higher order brain regions, instead highlighting their engagement of the ventral face network in a manner comparable with human faces.

## Supplementary Material

Supplementary Material

## Data Availability

All (pseudonymized) SEEG data are publicly available athttps://osf.io/p57ra/
