# Supplementary material for: The neural basis of face pareidolia with human intracerebral recordings
Source: Imaging Neurosci (Camb). 2025 Mar 31;3:imag_a_00518. doi: 10.1162/imag_a_00518 (PMC12319871; doi:10.1162/imag_a_00518)
Supplement: Supplementary Material [file imag_a_00518-supp.pdf]

SUPPLEMENTARY MATERIAL

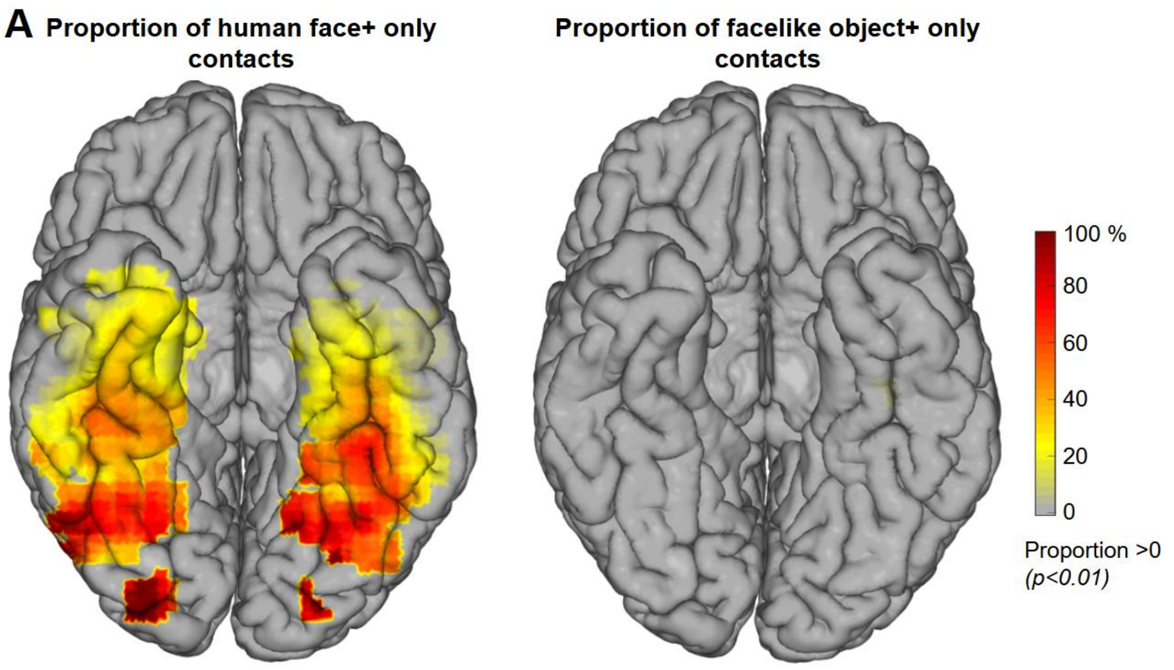

Fig. S1. Local proportions of human face+ only (contacts showing selective responses for human faces but not for facelike objects) and facelike object+ only contacts. The maps show the local proportion of the human face+ only and facelike object+ only contacts relative to recorded contacts across VOTC. The local proportions are computed in 15 x 15 mm voxels for x (medio-lateral) and y (postero-anterior) Talairach dimensions respectively, collapsing contacts across z-dimension (inferior-superior) for visualization. Only voxels with a proportion of selective contacts significantly above zero ($p < .01$, percentile bootstrap) are displayed.

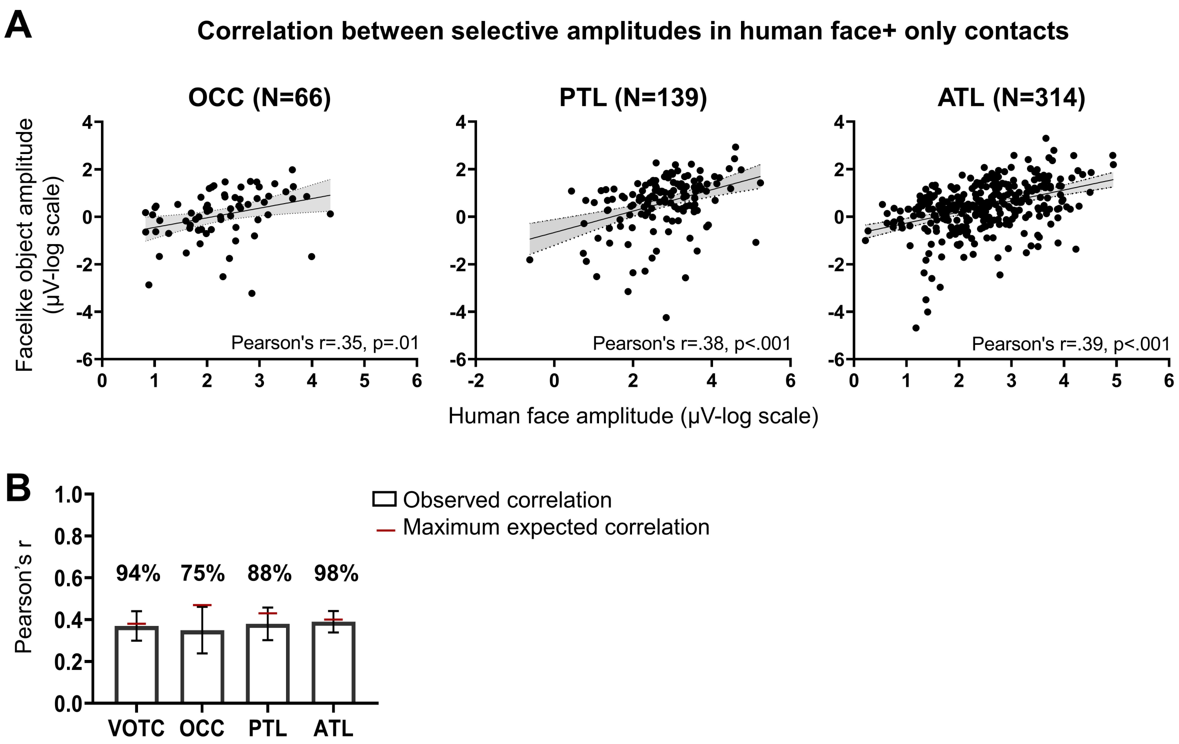

Fig. *S2*. Functional association between human face and facelike object responses in the human face+ only contacts. (A) Scatter plots presenting a linear relationship between the log-transformed amplitude responses for human face and facelike object in the human face+ only contacts in the three anatomical main regions (OCC, PTL, ATL). (B) Pearson's r values for the whole VOTC and each main region (as shown in bars) presented with the maximum expected correlation (MEC, as shown by red lines) considering the noise in each dataset. The percentages above each bar represent the ratio of actual correlation to the MEC, indicating the percentage of the maximum expected correlation achieved in each region.

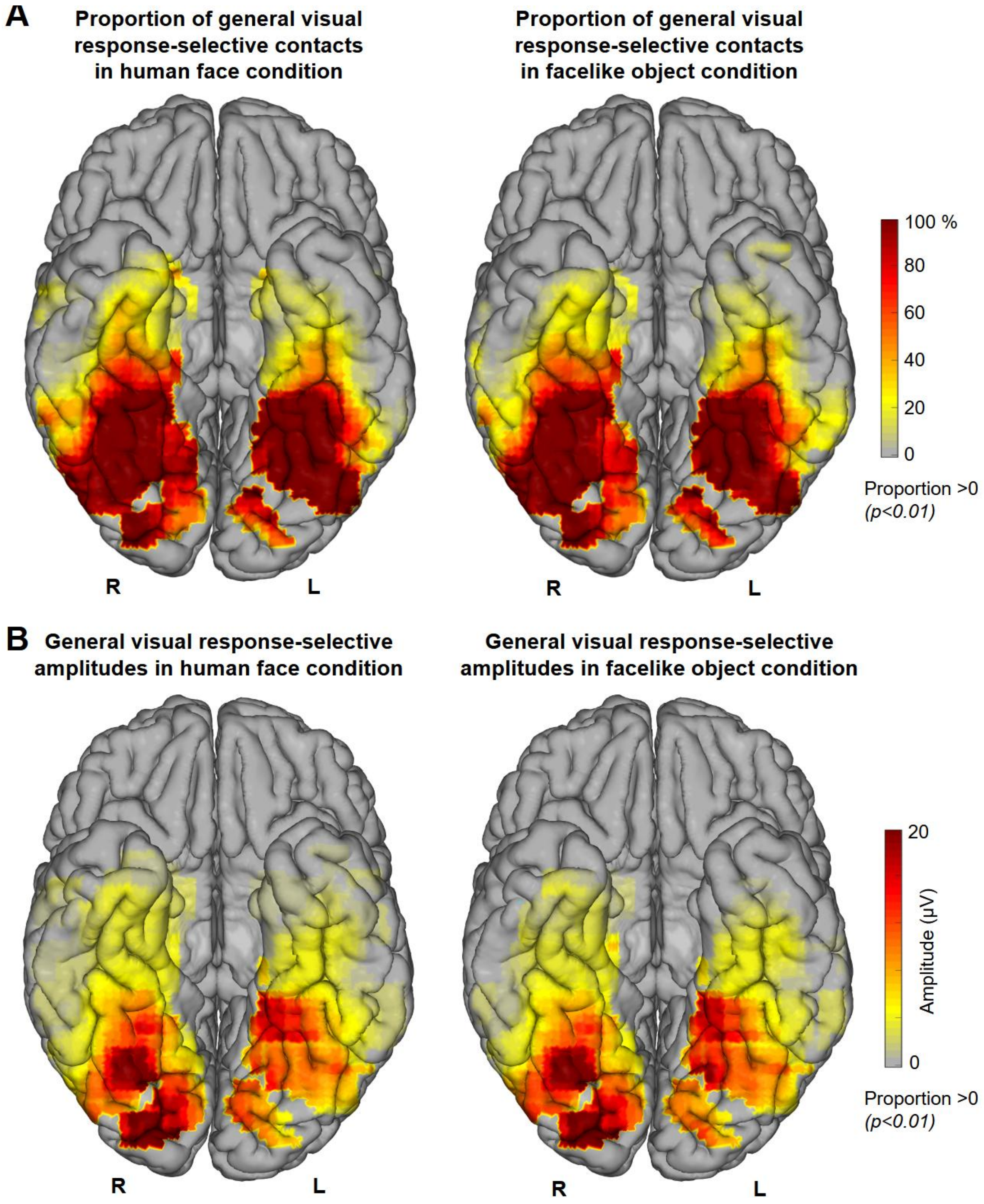

Fig. S3. Proportions and amplitudes of the general visual response-selective contacts in the human face and facelike object conditions. (A) Maps of local proportion of selective contacts relative to recorded contacts across VOTC for each condition. The local proportions are computed in 15 x 15 mm voxels for x (medio-lateral) and y (postero-anterior) Talairach dimensions respectively, collapsing

contacts across z-dimension (inferior-superior) for visualization. Only voxels with a proportion of selective contacts significantly above zero ($p < .01$, percentile bootstrap) are displayed. (B) General visual response-selective amplitudes in contacts where a significant general visual response was found in the human face and facelike object conditions. Amplitudes are quantified as the mean of the amplitudes across contacts within 15 x 15 mm voxels. Only voxels with a proportion of general visual response-selective contacts significantly above zero ($p < .01$, percentile bootstrap) are displayed.
